# Predictive value of the serum uric acid to high-density lipoprotein cholesterol ratio for culprit plaques in patients with acute coronary syndrome

**DOI:** 10.1186/s12872-024-03824-z

**Published:** 2024-03-13

**Authors:** Fuxue Deng, Fang Jia, Yang Sun, Lisha Zhang, Jie Han, Danni Li, Qiang Yang, Rongrong Hou, Wei Jiang

**Affiliations:** 1 Department of Cardiology, The Second Affiliated Hospital of Xi’an Jiaotong University, Xi’an, 710004 Shaanxi China; 2 Department of Endocrinology, The Second Affiliated Hospital of Xi’an Jiaotong University, Xi’an, 710004 Shaanxi China

**Keywords:** Optical coherence tomography, Acute coronary syndrome, High-density lipoprotein cholesterol, Uric acid, Uric acid to HDL cholesterol ratio

## Abstract

**Background:**

Hyperuricemia and low level of high-density lipoprotein cholesterol (HDL-C) are both risk factors for coronary artery disease (CAD). The uric acid to HDL-C ratio (UHR) has recently been identified as a new inflammatory and metabolic biomarker. However, the relationship between the UHR and coronary culprit plaques has not been fully investigated in patients with acute coronary syndrome (ACS).

**Methods:**

A total of 346 patients with ACS were enrolled in this study. Culprit lesion characteristics were assessed by optical coherence tomography (OCT). Logistic regression and linear correlation analyses were performed to assess the association between the UHR and culprit plaques. The predictive value of the UHR was investigated by receiver operating characteristic (ROC) curve analysis.

**Results:**

The percentages of typical culprit plaques, including ruptures, erosions and thrombi, were greater in the high-UHR subgroup than those in the low-UHR subgroup. A positive relationship was also found between the UHR and diameter stenosis (*r* = 0.160, *P* = 0.003) and between the UHR and area stenosis (*r* = 0.145, *P* = 0.007). The UHR was found to be independently associated with plaque rupture, erosion and thrombus. Furthermore, ROC analysis suggested that the UHR had a better predictive value than low-density lipoprotein cholesterol.

**Conclusions:**

An elevated UHR level was independently related to the occurrence rate of culprit plaques. The UHR is a simple and easily acquired parameter for detecting culprit plaques in patients with ACS.

**Supplementary Information:**

The online version contains supplementary material available at 10.1186/s12872-024-03824-z.

## Background

Uric acid (UA) is the end product of purine nucleotide metabolism. Previous studies have shown that hyperuricemia is positively correlated with coronary artery disease (CAD), hypertension, atrial fibrillation, heart failure, and even diabetes mellitus (DM) [1]. Moreover, hyperuricemia independently predicts an increased risk of major cardiovascular adverse events (MACEs) and mortality not only in elderly patients but also in young adults with acute coronary syndrome (ACS) [2, 3]. Elevated serum UA promotes an inflammatory response, oxidative stress, and endothelial dysfunction in patients with ACS [4]. Hyperuricemia is also associated with the progression of atherosclerosis [5].

High-density lipoprotein cholesterol (HDL-C) is traditionally considered the “good cholesterol” because it removes and transports excess cholesterol from the periphery to the liver [6]. Low HDL-C levels are a risk factor for atherosclerotic CAD [7]. Recently, the UA to HDL-C ratio (UHR) has been recognized as a biomarker for metabolic disorders and inflammation in many diseases, including CAD [8, 9]. Culprit plaques, such as plaque rupture, erosion and thrombus, are fundamental to the pathogenesis of ACS [10]. However, the association between the UHR and culprit plaques has not been explored. In the present study, we aimed to investigate the association between UHR and culprit plaques by optical coherence tomography (OCT) in patients with ACS.

## Patients and methods

### Study population

Patients with ACS who underwent percutaneous coronary intervention (PCI) were retrospectively recruited from the Second Affiliated Hospital of Xi’an Jiaotong University between March 2019 and September 2021. OCT was performed to identify the culprit lesions of the target vessels during PCI. The main exclusion criteria were: cardiogenic shock, end-stage renal disease, serious liver dysfunction, allergy to contrast media, left main disease, chronic total occlusion, and heavily calcified or tortuous vessels, which can render OCT examination difficult. Patients with poor OCT imaging quality and in-stent restenosis were also excluded.

The diagnosis and classification of ACS, which includes ST-elevation myocardial infarction (STEMI), non-ST-elevation myocardial infarction (NSTEMI) and unstable angina, were based on clinical symptoms, electrocardiogram findings and laboratory test results according to the guidelines. Blood samples were collected in tubes containing 0.1% EDTA after the patients were hospitalized. The baseline characteristics, medical history and personal habits of the patients were obtained. The laboratory parameters were tested according to standard protocols. The UHR was calculated as the ratio of UA (mg/dl) to HDL-C (mg/dl) and is expressed as a percentage (%).

This retrospective study was conducted in accordance with the Strengthening the Reporting of Observational Studies in Epidemiology (STROBE) guidelines for cross-sectional studies. The study protocol was approved by the Ethics Committee of the Second Affiliated Hospital of Xi’an Jiaotong University, and this study was also conducted according to the principles of the Helsinki Declaration II. All patients provided written informed consent.

### Culprit lesion identification

The transradial access was the preferred approach for coronary angiography and PCI. The culprit vessel was identified according to the results of angiography and electrocardiogram, and an OCT examination was conducted to determine the culprit lesion in the target vessels. All patients received standard dual antiplatelet medication according to guidelines, that is, initial oral treatment with aspirin (300 mg) and ticagrelor (180 mg) or clopidogrel (300 mg) followed by aspirin (100 mg) daily and ticagrelor (90 mg) twice daily or clopidogrel daily (75 mg). An intravascular infusion of 100 IU/kg of heparin was administered prior to PCI. After the drug-eluted stent was implanted, glycoprotein IIb/IIIa receptor inhibitors were administered intravenously when necessary.

### OCT image acquisition

Intravascular OCT images were obtained using a commercial frequency-domain OCT system (OCT Mobile Dragonfly, St. Jude Medical/Abbott, St. Paul, MN, USA). An OCT image catheter was advanced distal to the culprit lesion after flushing with 0.2 mg of nitroglycerin. A virtually blood-free environment was created by continuous injection of the contrast agent, and the OCT catheter was immediately pulled back manually or automatically at a rate of 20 mm/s. The total length of the OCT pullback was 75 mm during every single examination.

### OCT image analysis

All OCT images were analyzed on a St. Jude OCT Review Workstation by three independent physicians who were blinded to the study conditions. The culprit plaque was classified according to established criteria as we previously described [11]. Fibrous plaque features with homogeneous and highly backscattering regions. Lipid-rich plaques have low signal regions and diffuse borders. The fibrous cap thickness (FCT) of fibrous plaques or lipid-rich plaques was measured in triplicate, and the average value was calculated. The lipid arc was measured at the largest lipid region across the entire lesion. Thin-cap fibroatheroma (TCFA) was defined as a lipid-rich plaque with the thinnest part of the fibrous cap (< 65 μm). Plaque rupture was defined as disruption of the fibrous cap with cavity formation. Plaque erosion was identified as a thrombus attached to an irregular but intact luminal surface. Thrombi were classified as white, red, or mixed type according to the light scattering rate. Calcification was characterized by sharply delineated and low-backscattering heterogeneous regions. The arc, depth, and length of the calcification lesion were measured three times. Macrophages were defined as signal-rich regions with heterogeneous backward shadows. Cholesterol crystals were recognized as linear and highly backscattering regions within a plaque, especially in fibrous plaques. The minimal lumen area (MLA) and minimal lumen diameter (MLD) were automatically calculated along the length of the culprit lesion. The percentage of area of stenosis (AS) and diameter of stenosis (DS) were also recorded at the same time.

### Statistical analysis

Continuous data are presented as the mean ± standard deviation (SD). One-way analysis of variance (ANOVA) was used to compare groups. Categorical data are presented as frequencies and percentages (%), and the results were compared using the chi-square test or Fisher’s exact test. Correlations among variables were measured using the Spearman’s rank test or Pearson’s test. Univariate and multivariate logistic regression analyses were conducted to determine associations between culprit lesions and target variables. The predictive values of variables were examined by receiver operating characteristic (ROC) curve analysis and the area under the curve (AUC) was calculated. A two-tailed *P* < 0.05 was considered to indicate statistical significance. Analyses were conducted using SPSS 26.0 statistical software (SPSS Inc, Chicago, IL, USA).

## Results

### Baseline demographic and clinical characteristics

**Fig. 1:**
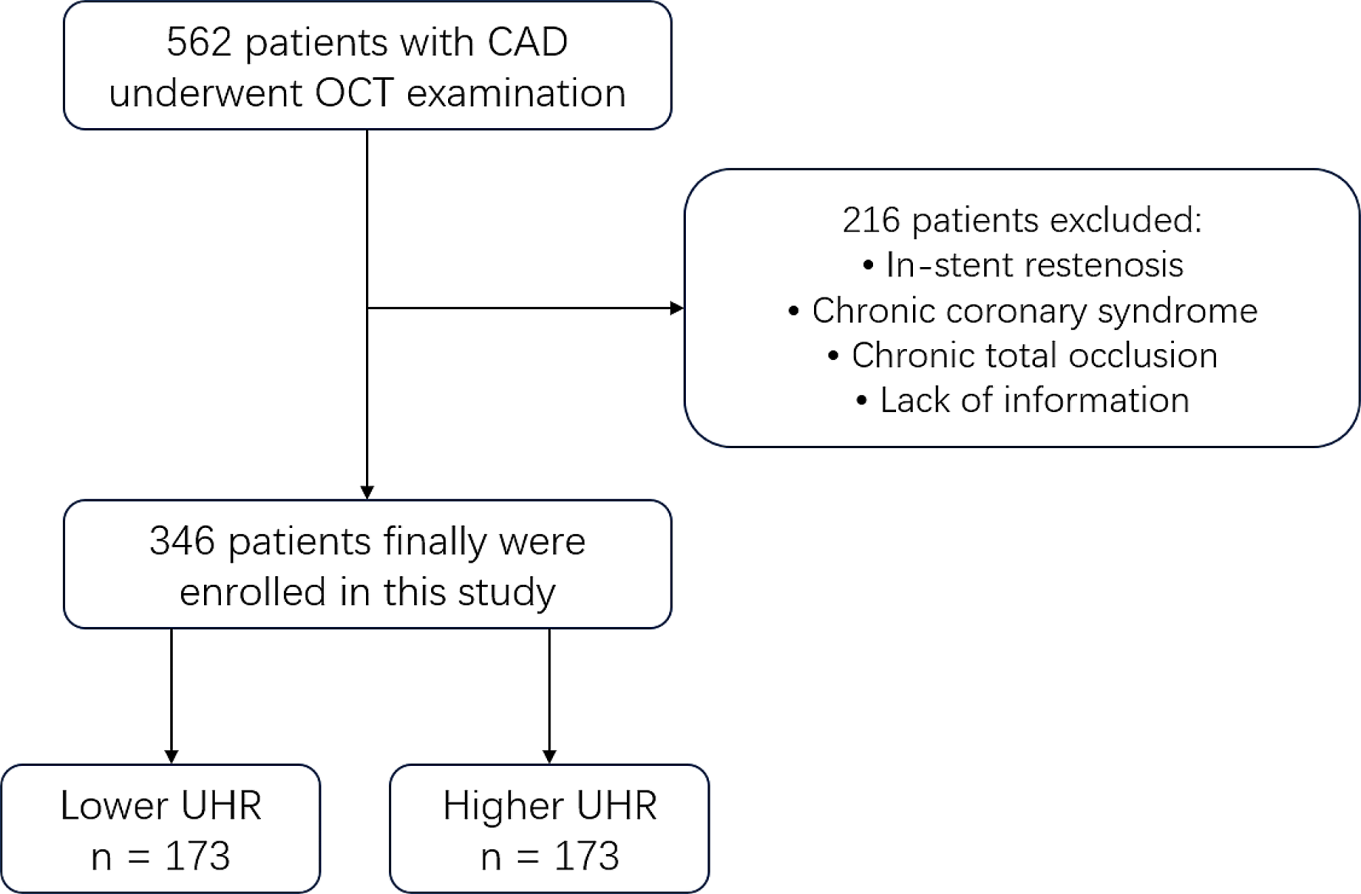
The flow chart of the study. CAD, coronary artery disease; OCT, optical coherence tomography; UHR, UA to HDL-C ratio

Between March 2019 and September 2021, a total of 562 patients with ACS were recruited and underwent OCT examination. A total of 216 patients were excluded due to different reasons for exclusion. Finally, 346 patients with ACS were enrolled in this study and the samples were divided into two groups by the median of UHR (13.64%). The flow chart of this study is shown in Fig. 1. The average age of the patients was 58.83 years, and 76.9% of them were male. The percentages of patients with different types of ACS were compared, and no significant differences were found (*P* = 0.103). Significant differences were found in the proportion of males, current smoking status, alcohol consumption status, age, number of smoking years, hemoglobin (Hb) level, thyroid stimulating hormone (TSH) level, triglyceride (TG) level, HDL-C level, UHR, very low-density lipoprotein cholesterol (VLDL) level, estimated glomerular filtration rate (eGFR), apolipoprotein (Apo) A1 level and lipoprotein(a) [Lp(a)] level (Table 1). Furthermore, the proximal part of the left anterior descending artery accounted for the majority of the culprit vessels.

**Table 1:** Baseline clinical and OCT characteristics

	All (*n* = 346)	Lower UHR (*n* = 173)	Higher UHR (*n* = 173)	P value
Age, years	58.83 ± 11.46	61.82 ± 10.71	55.85 ± 11.43	< 0.001
Male, n (%)	266 (76.9)	110 (63.6)	156 (90.2)	< 0.001
Medical history, n (%)				
Atrial fibrillation	11 (3.2)	7 (4.0)	4 (2.3)	0.358
Hypertension	190 (54.9)	94 (54.3)	96 (55.5)	0.829
Diabetes mellitus	84 (24.3)	40 (23.1)	44 (25.4)	0.616
CAD	155 (44.8)	78 (45.1)	77 (44.5)	0.914
Previous PCI	43 (12.4)	19 (11.0)	24 (13.9)	0.415
Stroke	14 (4.0)	9 (5.2)	5 (2.9)	0.275
Alcohol consumption	54 (15.6)	20 (11.6)	34 (19.7)	0.038
Current smoking	145 (41.9)	53 (30.6)	92 (53.2)	< 0.001
Smoking years	13.74 ± 16.20	10.50 ± 15.64	16.95 ± 16.16	< 0.001
Statins, n (%)	228 (65.9)	107 (61.8)	121 (69.9)	0.112
UA-lowering drugs, n (%)	30 (8.7)	14 (8.1)	16 (9.2)	0.862
Febuxostat	19 (5.5)	8 (4.6)	11 (6.4)	
Benzbromarone	9 (2.6)	5 (2.9)	4 (2.3)	
Allopurinol	2 (0.6)	1 (0.6)	1 (0.6)	
Diagnosis, n (%)				0.103
Unstable angina	185 (53.5)	101 (58.4)	84 (48.6)	
NSTEMI	110 (31.8)	46 (26.6)	64 (37.0)	
STEMI	51 (14.7)	26 (15.0)	25 (14.5)	
Laboratory results, mean ± SD				
WBC, ×10^9^/L	6.98 ± 2.42	6.88 ± 2.62	7.08 ± 2.21	0.459
Hb, g/L	141.82 ± 17.07	139.323 ± 16.27	144.31 ± 17.52	0.006
HbA1c, %	6.49 ± 1.69	6.41 ± 1.36	6.56 ± 1.96	0.442
NT-proBNP, pg/ml	599.78 ± 1756.07	522.21 ± 1439.95	678.76 ± 2029.41	0.414
Hs-cTnI, pg/ml	316.80 ± 959.76	297.73 ± 921.31	335.52 ± 998.54	0.724
CK-MB, U/L	25.76 ± 35.76	29.27 ± 43.55	22.33 ± 25.69	0.074
Glucose, mmol/L	5.99 ± 2.21	5.99 ± 2.53	5.98 ± 1.88	0.961
Creatine, µmol/L	74.65 ± 50.80	71.90 ± 69.31	77.41 ± 18.93	0.314
eGFR, ml/min/1.73m^2^	100.15 ± 25.18	103.37 ± 27.33	96.93 ± 22.44	0.017
TC, mmol/L	3.79 ± 1.00	3.87 ± 0.94	3.71 ± 1.05	0.148
TG, mmol/L	1.80 ± 1.43	1.43 ± 0.69	2.17 ± 1.83	< 0.001
UA, mg/dl	5.47 ± 1.41	4.55 ± 0.95	6.38 ± 1.19	< 0.001
HDL-C, mg/dl	41.06 ± 10.16	47.37 ± 9.70	34.76 ± 5.76	< 0.001
UHR (%)	14.36 ± 5.77	9.92 ± 2.37	18.80 ± 4.64	< 0.001
LDL-C, mmol/L	2.19 ± 0.86	2.24 ± 0.87	2.15 ± 0.86	0.334
VLDL-C, mmol/L	0.54 ± 0.47	0.42 ± 0.25	0.66 ± 0.60	< 0.001
Apo A1, g/L	1.24 ± 0.26	1.37 ± 0.26	1.11 ± 0.18	< 0.001
Apo B, g/L	0.84 ± 0.27	0.83 ± 0.26	0.86 ± 0.27	0.292
Lp(a), mg/dl	21.90 ± 27.19	25.36 ± 29.83	18.43 ± 23.85	0.018
TSH, µIU/ml	3.26 ± 4.35	3.89 ± 5.95	2.63 ± 1.42	0.010
D-dimer, µg/ml	485.62 ± 1098.93	492.01 ± 918.72	479.19 ± 1257.11	0.914
LVEF, %	63.14 ± 7.94	64.21 ± 7.03	62.06 ± 8.65	0.017
Culprit vessels, n (%)				0.613
LAD	237 (68.5)	123 (71.1)	114 (65.9)	
LCX	43 (12.4)	20 (11.6)	23 (13.3	
RCA	66 (19.1)	30 (17.3)	36 (20.8)	
Lesion site, n (%)				0.220
Proximal	209 (60.4)	103 (59.5)	106 (61.3)	
Middle	121 (35.0)	65 (37.6)	56 (32.4)	
Distal	16 (4.6)	5 (2.9)	11 (6.4)	
Stents, n (%)				0.240
0	111 (32.1)	61 (35.3)	50 (28.9)	
1	141 (40.8)	72 (41.6)	69 (39.9)	
2	60 (17.3)	29 (16.8)	31 (17.9)	
3	27 (7.8)	9 (5.2)	18 (10.4)	
>3	7 (2.0)	2 (1.2)	5 (2.9)	
Plaque morphology, n (%)				
Plaque rupture	95 (27.5)	36 (20.8)	59 (34.1)	0.006
Plaque erosion	68 (19.7)	26 (15.0)	42 (24.3)	0.054
Calcified nodule	12 (3.5)	7 (4.0)	5 (2.9)	0.170
Plaque type, n (%)				
Thrombus	118 (34.1)	45 (26.0)	73 (42.2)	0.002
Red thrombus	6 (1.7)	2 (1.2)	4 (2.3)	
White thrombus	71 (20.5)	30 (17.3)	41 (23.7)	
Mixed thrombus	41 (11.8)	13 (7.5)	28 (16.2)	
TCFA	23 (6.6)	12 (6.9)	11 (6.4)	0.234
Fibrous plaque	161 (46.5)	79 (45.7)	82 (47.4)	0.441
FCT of fibrous plaque, µm	981.62 ± 330.96	980.39 ± 362.75	982.83 ± 298.57	0.963
Calcification	89 (25.7)	55 (31.8)	34 (19.7)	0.023
angle, °	190.34 ± 112.45	179.23 ± 107.91	206.86 ± 118.51	0.264
thickness, mm	11.01 ± 93.72	17.62 ± 120.52	0.91 ± 0.29	0.422
length, mm	23.40 ± 13.11	20.68 ± 9.60	27.27 ± 16.30	0.043
Lipid-rich plaque	88 (25.4)	39 (22.5)	49 (28.3)	0.172
FCT, µm	147.31 ± 178.09	121.55 ± 88.65	166.75 ± 222.08	0.227
Lipid arc, °	204.35 ± 69.60	215.80 ± 60.03	195.54 ± 75.55	0.168
Cholesterol crystal	151 (43.6)	71 (41.0)	80 (46.2)	0.355
Micro-vessel	72 (20.8)	33 (19.1)	39 (22.5)	0.265
Macrophage	87 (25.1)	44 (25.4)	43 (24.9)	0.829
Quantitative of target vessel				
MLA, mm^2^	2.14 ± 1.37	2.23 ± 1.34	2.03 ± 1.40	0.192
MLD, mm	1.58 ± 0.46	1.61 ± 0.45	1.55 ± 0.48	0.184
Diameter stenosis, %	45.23 ± 12.28	43.40 ± 12.60	47.06 ± 11.71	0.005
Area stenosis, %	68.95 ± 14.60	66.79 ± 15.67	71.11 ± 13.13	0.006
Reference vessel diameter, mm	2.69 ± 0.68	2.64 ± 0.62	2.73 ± 0.73	0.242
Post-stent MLA, mm^2^	5.01 ± 1.96	5.16 ± 2.34	4.89 ± 1.57	0.334

OCT, optical coherence tomography; UHR, UA to HDL-C ratio; CAD, coronary artery disease; PCI, percutaneous coronary intervention; UA, uric acid; NSTEMI, non-ST segment elevated myocardial infarction; STEMI, ST segment elevated myocardial infarction; SD, standard deviation; WBC, white blood cell; Hb, hemoglobin; HbA1c, glycosylated hemoglobin; NT-proBNP, N-terminal B-type natriuretic peptide; Hs-cTnI, high-sensitivity cardiac troponin I; CK-MB, creatine kinase-MB; eGFR, estimated glomerular filtration rate; TC, total cholesterol; TG, total triglycerides; HDL-C, high-density lipoprotein cholesterol; LDL-C, low-density lipoprotein cholesterol; VLDL-C, very low-density lipoprotein cholesterol; Apo, apolipoprotein; Lp(a), lipoprotein (a); TSH, thyroid stimulating hormone; LVEF, left ventricular ejection fraction; LAD, left anterior descending artery; LCX, left circumflex artery; RCA, right coronary artery; TCFA, thin-cap fibroatheroma; FCT, fibrous cap thickness; MLA, minimal lumen area; MLD, minimal lumen diameter

### OCT findings in different groups

**Fig. 2:**
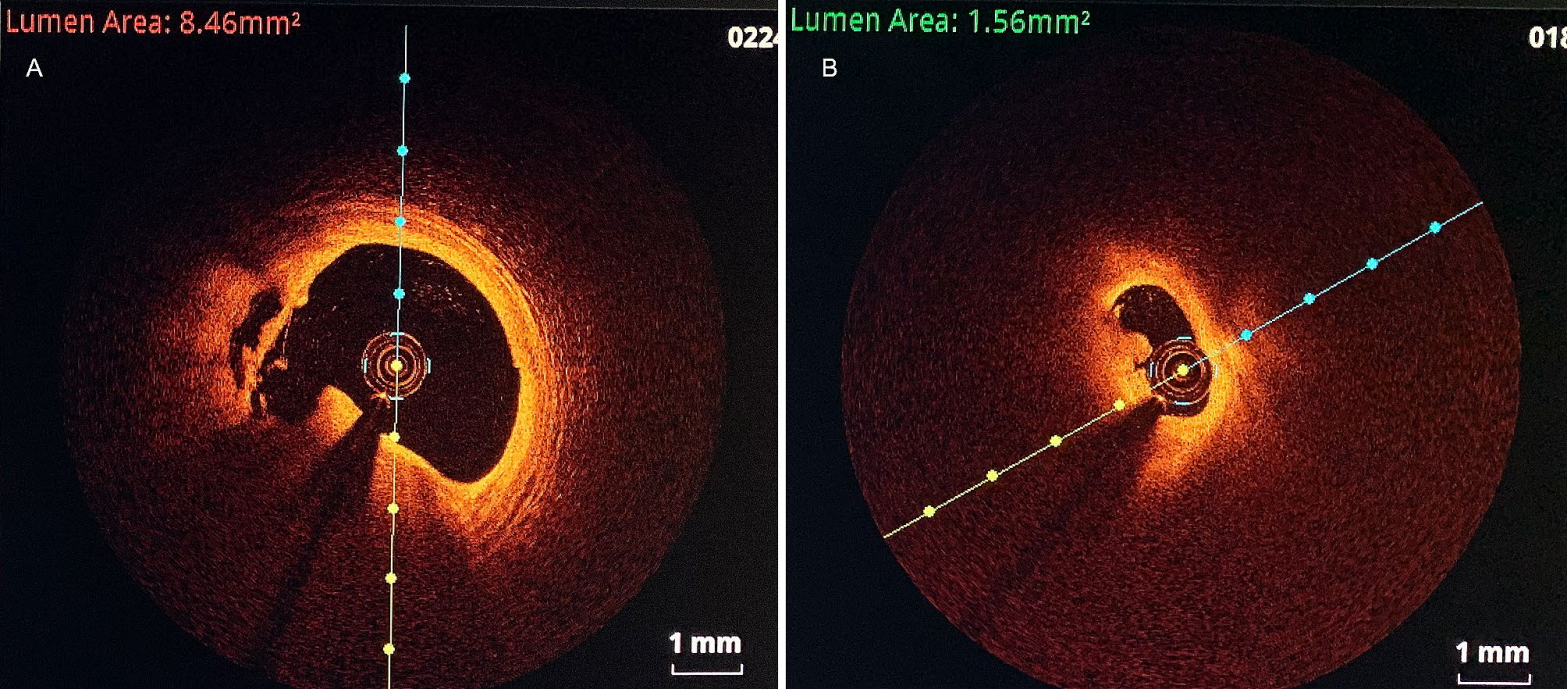
The representative images of plaque rupture and erosion with thrombus by OCT. (**A**) Plaque rupture and white thrombus. (**B**) Plaque erosion and white thrombus. The lumen area indicated in the upper of the OCT images

Representative OCT images of plaque rupture and erosion with thrombi are shown in Fig. 2. The percentages of typical culprit plaques, including rupture, erosion and thrombus, were greater in the high-UHR group than those in the low-UHR group (Table 1). The percentage of plaque rupture was also greater than that of plaque erosion and calcified nodules (25.4% versus 17.7% versus 3.5%). Furthermore, the percentage of white thrombi was greater than that of red or mixed thrombi. No significant differences were found in other plaque types, such as fibrous plaques, lipid plaques, cholesterol crystals and macrophages.

### Correlation of the UHR with plaque parameters

To further investigate the associations between the UHR and plaque parameters, a series of correlation studies were performed (Table 2). Among these parameters, white blood cells (*r* = 0.143, *P* = 0.008), Hb (*r* = 0.151, *P* = 0.005), and TG (*r* = 0.298, *P* < 0.001) was positively correlated with the UHR. A positive relationship was also found between UHR and DS (*r* = 0.160, *P* = 0.003) and between the UHR and AS (*r* = 0.145, *P* = 0.007). In addition, the age (*r* = -0.311, *P* < 0.001), eGFR (*r* = -0.161, *P* = 0.003), TC (*r* = -0.106, *P* = 0.049), Apo A1 (*r* = -0.556, *P* < 0.001), Lp(a) (*r* = -0.155, *P* = 0.004), TSH (*r* = -0.152, *P* = 0.006) and left ventricular ejection fraction (LVEF) (*r* = -0.184, *P* = 0.001) were negatively correlated with the UHR.

**Table 2:** Correlations of the UHR with biochemical parameters

	UHR	
	r	P value
Age	-0.311	< 0.001
WBC	0.143	0.008
Hb	0.151	0.005
HbA1c	0.037	0.507
NT-proBNP	0.072	0.185
Hs-cTnI	0.002	0.967
Glucose	0.024	0.706
Crea	0.076	0.157
eGFR	-0.161	0.003
TC	-0.106	0.049
TG	0.298	< 0.001
LDL-C	-0.071	0.188
VLDL-C	0.274	< 0.001
Apo A1	-0.556	< 0.001
Apo B	0.062	0.253
Lp(a)	-0.155	0.004
TSH	-0.152	0.006
D-dimer	-0.023	0.670
LVEF	-0.184	0.001
MLA	-0.072	0.179
MLD	-0.072	0.181
AS	0.145	0.007
DS	0.160	0.003
Calcification angle	0.208	0.053
Calcification thickness	-0.074	0.497
Calcification length	0.272	0.015
FCT of lipid plaque	-0.004	0.969
Angle of lipid plaque	-0.126	0.230
Fibrosus thickness	-0.014	0.866

UHR, UA to HDL-C ratio; r, correlation coefficient; WBC, white blood cell; Hb, hemoglobin; HbA1c, glycosylated hemoglobin; NT-proBNP, N-terminal B-type natriuretic peptide; Hs-cTnI, high-sensitivity cardiac troponin I; Crea, creatine; eGFR, estimated glomerular filtration rate; TC, total cholesterol; TG, total triglycerides; LDL-C, low-density lipoprotein cholesterol; VLDL-C, very low-density lipoprotein cholesterol; Apo, apolipoprotein; Lp(a), lipoprotein (a); TSH, thyroid stimulating hormone; LVEF, left ventricular ejection fraction; MLA, minimal lumen area; MLD, minimal lumen diameter; AS, are stenosis; DS, diameter stenosis; FCT, fibrous cap thickness

### Univariate and multivariate logistic regression analyses

Univariate logistic regression analysis revealed that the UHR (continuous variable or categorical variable) was closely related with plaque rupture. After adjusting for different confounding parameters, including age, sex, medical history, smoking status, alcohol consumption, statins and UA-lowering drugs in four different models, the UHR was found to be independently associated with plaque rupture. Furthermore, the UHR was divided into four quartiles in each model and multivariate logistic regression analysis was also performed. The UHR was also significantly associated with the odds of plaque rupture (Table S1). Similar results were found for erosion (Table S2) and thrombi (Table S3).

### ROC curve analysis

**Fig. 3:**
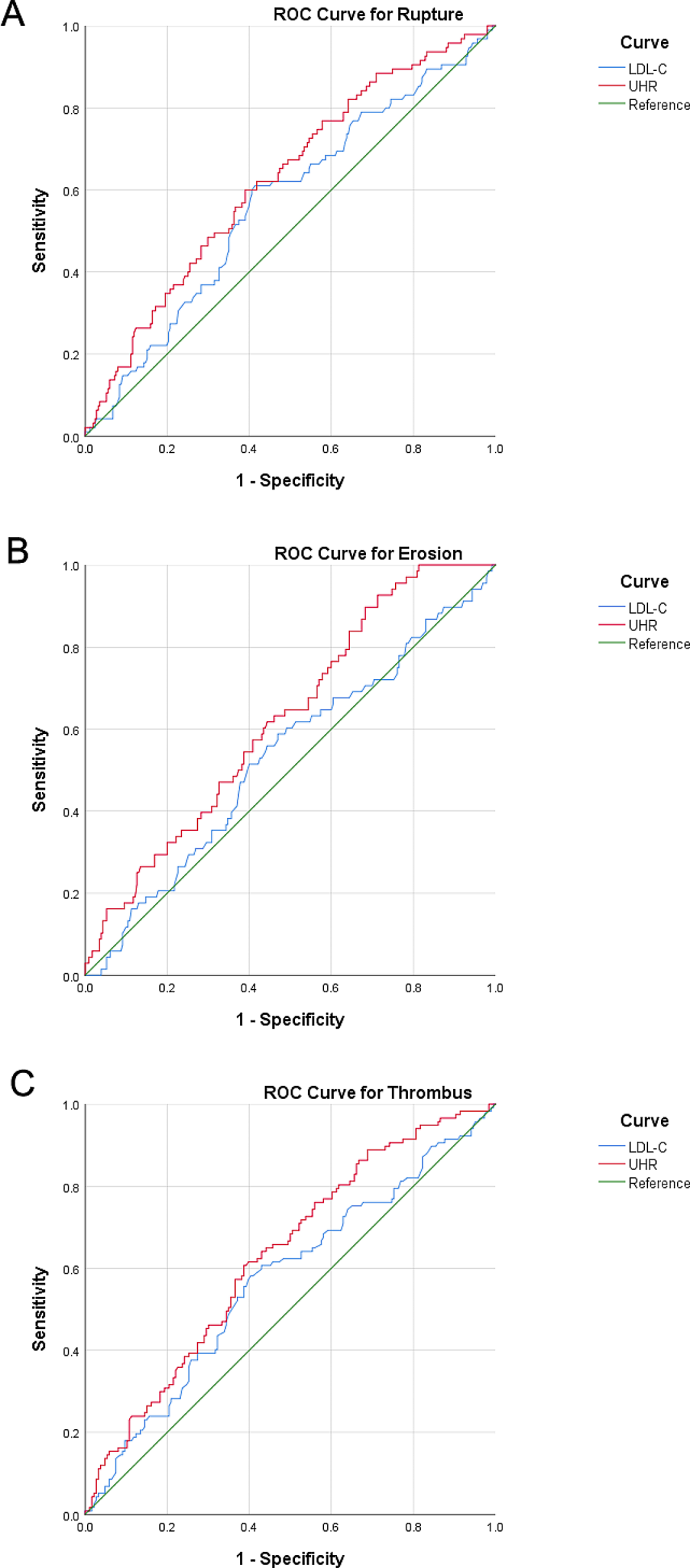
Receiver operating characteristic (ROC) curve for plaque rupture (**A**), erosion (**B**) and thrombus (**C**). UHR, UA to HDL-C ratio; LDL-C, low-density lipoprotein cholesterol

To investigate the predictive capacity of the UHR for culprit plaques, a series of ROC curve analyses were conducted. In plaque rupture group, the AUC value for the UHR was 0.625 (95% CI: 0.560–0.690, *P* < 0.001), which was greater than that for LDL-C [0.568 (95% CI: 0.501–0.636, *P* = 0.568)] (Fig. 3A). Similar results were found for plaque erosion and thrombus. In the plaque erosion group, the AUC was 0.624 (95% CI: 0.554–0.695, *P* = 0.002) for UHR, and 0.530 (95% CI: 0.452–0.609, *P* = 0.445) for LDL-C (Fig. 3B). In the thrombus group, the AUC was 0.631 (95% CI: 0.567–0.694, *P* < 0.001) for the UHR, and 0.571 (95% CI: 0.504–0.638, *P* = 0.037) for LDL-C (Fig. 3C).

## Discussion

In the present study, the relationship between the UHR and the characteristics of culprit plaques determined by OCT in patients with ACS was investigated. We found that the UHR, a novel parameter combining UA and HDL-C, was significantly associated with culprit plaques and showed excellent predictive value for culprit plaques and thrombi in patients with ACS. To the best of our knowledge, this is the first study concerning the relationship between the UHR and culprit plaques in patients with ACS.

The concentration of UA is controlled by xanthine oxidase. A high level of UA is associated with short- or long-term adverse MACEs and mortality in patients with ACS who are undergoing PCI [12]. For each 1 mg/dL increase in UA level, the risk of death from atherosclerotic disease increased by 48% in men and 126% in women [13]. A cross-sectional study with a sample of 1 947 hospitalized patients with DM showed that the serum UA levels were closely associated with metabolic syndrome but not with carotid atherosclerosis [14]. However, in another study, a high level of UA was associated with metabolic syndrome and was an independent risk factor for carotid atherosclerosis in patients with DM [15]. A linear association between high UA variability and the development of CAD was found in a retrospective cross-sectional study, and patients with high levels of UA were older and had higher mortality from CAD [16]. Furthermore, hyperuricemia was found to be an independent risk factor for a high atherogenic index of plasma levels [17].

Regarding the possible mechanism, hyperuricemia significantly promotes the development of atherosclerotic plaques, downregulates the protein levels of inflammatory genes involved in signaling pathway, promotes the formation of foam cells, and increases lipid peroxidation in macrophages treated with oxidized LDL [18, 19]. A previous study also indicated that UA levels are positively associated with inflammatory cytokines, such as interleukin (IL)-6, IL-1β, C-reactive protein, and tumor necrosis factor, and may subsequently induce metabolic abnormalities [20, 21]. Overall, UA represents one of the indicators of oxidative homeostasis and plays a central role in the development of coronary plaques [22].

As an important component of lipid metabolism in the human body, HDL-C is composed of cholesterol, triglycerides, phospholipids and apolipoproteins, especially Apo A1. It has been demonstrated that HDL-C plays an important role in promoting cholesterol efflux in arterial wall cells to the liver and preventing arteriosclerosis in circulation through its anti-inflammatory and antioxidant effects [23, 24]. Although there is controversy regarding the relationship between HDL-C and CAD, the metabolism of HDL-C is far more complex and HDL-C itself remains a standard marker of CAD risk [25, 26]. More mechanistic and clinical studies are needed in the future to explore the exact relationship between HDL-C and cardiovascular risk.

Recently, the UHR has attracted clinical attention clinically as a novel biomarker for different diseases. A high UHR was found to be positively associated with the incidence of ischemic heart diseases without DM [8]. The UHR could serve as a promising predictor of diabetes control in men with DM [27]. Other studies have also shown that UA and HDL-C may interact with each other and have synergistic effects on atherosclerosis and CAD [28, 29]. However, no previous study has explored the relationship between UHR status and coronary artery culprit plaques in patients with ACS. In this study, we reported that a high UHR is associated with a greater incidence of plaque rupture and erosion, followed by the formation and presentation of a thrombus. This may be related to the high concentration of UA and low concentration of HDL-C, as we have shown in the manuscript. These synergistic adverse effects on CAD may be mediated through oxidative damage to endothelial cells and inflammation. It is also worth noting that there was no difference in the UHR level among patients with unstable angina, NSTEMI or STEMI. However, there may be differences in patients with ACS with those with chronic coronary syndrome if these patients are compared.

ACS mostly arises from the rupture or erosion of a culprit plaque [30, 31]. LDL-C is an independent predictor of culprit plaques. In the present study, we compared the performance of the UHR with that of LDL-C, and found that the UHR had a better AUC than LDL-C. In this study, we also predicted the presence of culprit plaques or thrombi in patients with ACS by simply calculating the index of the UHR. For patients with a greater UHR, we have more reasons to perform OCT examinations via PCI to specify and treat culprit plaques.

**Study limitations**.

This study has several limitations. First, this retrospective study included only has a small number of patients with ACS. Second, the blood samples were acquired when patients were admitted to the hospital, and clinical results were obtained only once. Third, the OCT examinations were mainly conducted in the culprit vessel according to coronary angiography results instead of all three coronary vessels. The need for good-quality images in OCT excluded distal lesion and high-risk patients, such as those with tortuous and high-calcified vessels, which made this study population too selected. Fourth, the MACE incidence and follow up of the patients were not further investigated. More work should be done and more patients and more vessels should be studied in the future.

**Conclusions**.

We found that plaque rupture, erosion and thrombus were more prevalent in the high-UHR group than in the low-UHR group, and the UHR could serve as an independent predictor for culprit or culprit plaques in patients with ACS.

### Electronic supplementary material

Below is the link to the electronic supplementary material.

Supplementary Material 1

Supplementary Material 2

Supplementary Material 3

## Data Availability

The datasets used and/or analyzed during the current study are available from the corresponding author on reasonable request.

## Abbreviations

HDL-C: high-density lipoprotein cholesterol
CAD: coronary artery disease
UA: uric acid
UHR: the uric acid to HDL-C ratio
ACS: acute coronary syndrome
OCT: optical coherence tomography
ROC: receiver operator characteristic
AUC: area under the curve
LDL-C: low-density lipoprotein cholesterol
DM: diabetes mellitus
MACEs: major cardiovascular adverse events
PCI: percutaneous coronary intervention
STEMI: ST-elevation myocardial infarction
NSTEMI: non-ST-elevation myocardial infarction
FCT: fibrous cap thickness
TCFA: thin-cap fibroatheroma
MLA: minimal lumen area
MLD: minimal lumen diameter
AS: area stenosis
DS: diameter stenosis
ANOVA: one-way analysis of variance
Hb: hemoglobin
TSH: thyroid stimulating hormone
TG: triglyceride
VLDL: very low-density lipoprotein cholesterol
eGFR: estimated glomerular filtration rate
Lp(a): lipoprotein(a)
IL: interleukin
