# Supplementary material for: Predictive value of the serum uric acid to high-density lipoprotein cholesterol ratio for culprit plaques in patients with acute coronary syndrome
Source: BMC Cardiovasc Disord. 2024 Mar 13;24:155. doi: 10.1186/s12872-024-03824-z (PMC10935860; doi:10.1186/s12872-024-03824-z)
Supplement: Supplementary file 1 — Supplementary Material 1 [file 12872_2024_3824_MOESM1_ESM.docx]

**Table S1. Logistic regression analysis of UHR for plaque rupture**

| **Variables** | **OR** | **95% CI** | ***P* value** |
| --- | --- | --- | --- |
| UA | 1.302 | 1.101-1.540 | 0.002 |
| HDL-C | 0.969 | 0.945-0.994 | 0.014 |
| LDL-C | 1.257 | 0.959-1.647 | 0.097 |
| UHR (continuous variable) | 1.074 | 1.031-1.119 | 0.001 |
| UHR (categorical variable) | 1.477 | 1.184-1.842 | 0.001 |
| **Model 1** | 1.409 | 1.108-1.792 | 0.005 |
| Q1 (4.77-10.25) | Reference |  |  |
| Q2 (10.26-13.64) | 2.018 | 0.945-4.308 | 0.070 |
| Q3 (13.65-17.39) | 2.264 | 1.069-4.797 | 0.033 |
| Q4 (17.40-38.83) | 3.672 | 1.767-7.630 | <0.001 |
| **Model 2** | 1.417 | 1.107-1.813 | 0.006 |
| Q1 (4.77-10.25) | Reference |  |  |
| Q2 (10.26-13.64) | 1.869 | 0.854-4.092 | 0.118 |
| Q3 (13.65-17.39) | 2.027 | 0.909-4.519 | 0.084 |
| Q4 (17.40-38.83) | 3.184 | 1.439-7.043 | 0.004 |
| **Model 3** | 1.405 | 1.097-1.801 | 0.007 |
| Q1 (4.77-10.25) | Reference |  |  |
| Q2 (10.26-13.64) | 2.026 | 0.902-4.549 | 0.087 |
| Q3 (13.65-17.39) | 2.183 | 0.953-5.002 | 0.065 |
| Q4 (17.40-38.83) | 3.250 | 1.425-7.410 | 0.005 |
| **Model 4** | 1.402 | 1.092-1.801 | 0.008 |
| Q1 (4.77-10.25) | Reference |  |  |
| Q2 (10.26-13.64) | 2.134 | 0.939-4.846 | 0.070 |
| Q3 (13.65-17.39) | 2.226 | 0.960-5.161 | 0.062 |
| Q4 (17.40-38.83) | 3.300 | 1.435-7.591 | 0.005 |

Model 1: UHR (categorical variable), age and gender.

Model 2: UHR (categorical variable), age, gender, atrial fibrillation, hypertension, diabetes mellitus and stroke.

Model 3: UHR (categorical variable), age, gender, atrial fibrillation, hypertension, diabetes mellitus, stroke, smoking and alcohol consumption.

Model 4: UHR (categorical variable), age, gender, atrial fibrillation, hypertension, diabetes mellitus, stroke, smoking, alcohol consumption, statins and UA-lowering drugs.

UA, uric acid; HDL-C, high-density lipoprotein cholesterol; LDL-C, low-density lipoprotein cholesterol; UHR, UA to HDL-C ratio; OR. odds ratio; CI, confidence interval.
