# Supplementary material for: Predictive value of the serum uric acid to high-density lipoprotein cholesterol ratio for culprit plaques in patients with acute coronary syndrome
Source: BMC Cardiovasc Disord. 2024 Mar 13;24:155. doi: 10.1186/s12872-024-03824-z (PMC10935860; doi:10.1186/s12872-024-03824-z)
Supplement: Supplementary file 2 — Supplementary Material 2 [file 12872_2024_3824_MOESM2_ESM.docx]

**Table S2. Logistic regression analysis of UHR for plaque erosion**

| **Variables** | **OR** | **95% CI** | ***P* value** |
| --- | --- | --- | --- |
| UA | 1.369 | 1.129-1.658 | 0.001 |
| HDL-C | 0.968 | 0.939-0.997 | 0.031 |
| LDL-C | 1.070 | 0.777-1.473 | 0.680 |
| UHR (continuous variable) | 1.083 | 1.033-1.135 | 0.001 |
| UHR (categorical variable) | 1.472 | 1.140-1.899 | 0.003 |
| **Model 1** | 1.385 | 1.051-1.824 | 0.021 |
| Q1 (4.77-10.25) | Reference |  |  |
| Q2 (10.26-13.64) | 3.135 | 1.231-7.988 | 0.017 |
| Q3 (13.65-17.39) | 3.418 | 1.347-8.673 | 0.010 |
| Q4 (17.40-38.83) | 4.297 | 1.701-10.858 | 0.002 |
| **Model 2** | 1.352 | 1.021-1.791 | 0.035 |
| Q1 (4.77-10.25) | Reference |  |  |
| Q2 (10.26-13.64) | 2.869 | 1.100-7.478 | 0.031 |
| Q3 (13.65-17.39) | 2.995 | 1.117-8.026 | 0.029 |
| Q4 (17.40-38.83) | 3.652 | 1.361-9.798 | 0.010 |
| **Model 3** | 1.380 | 1.040-1.831 | 0.025 |
| Q1 (4.77-10.25) | Reference |  |  |
| Q2 (10.26-13.64) | 3.841 | 1.285-11.479 | 0.016 |
| Q3 (13.65-17.39) | 2.886 | 0.931-8.951 | 0.066 |
| Q4 (17.40-38.83) | 3.500 | 1.106-11.076 | 0.033 |
| **Model 4** | 1.392 | 1.036-1.870 | 0.028 |
| Q1 (4.77-10.25) | Reference |  |  |
| Q2 (10.26-13.64) | 2.662 | 0.995-7.122 | 0.051 |
| Q3 (13.65-17.39) | 2.724 | 0.995-7.462 | 0.051 |
| Q4 (17.40-38.83) | 3.544 | 1.274-9.855 | 0.015 |

Model 1: UHR (categorical variable), age and gender.

Model 2: UHR (categorical variable), age, gender, atrial fibrillation, hypertension, diabetes mellitus and stroke.

Model 3: UHR (categorical variable), age, gender, atrial fibrillation, hypertension, diabetes mellitus, stroke, smoking and alcohol consumption.

Model 4: UHR (categorical variable), age, gender, atrial fibrillation, hypertension, diabetes mellitus, stroke, smoking, alcohol consumption, statins and UA-lowering drugs.

UA, uric acid; HDL-C, high-density lipoprotein cholesterol; LDL-C, low-density lipoprotein cholesterol; UHR, UA to HDL-C ratio; OR. odds ratio; CI, confidence interval.
