# Supplementary material for: Predictive value of the serum uric acid to high-density lipoprotein cholesterol ratio for culprit plaques in patients with acute coronary syndrome
Source: BMC Cardiovasc Disord. 2024 Mar 13;24:155. doi: 10.1186/s12872-024-03824-z (PMC10935860; doi:10.1186/s12872-024-03824-z)
Supplement: Supplementary file 3 — Supplementary Material 3 [file 12872_2024_3824_MOESM3_ESM.docx]

**Table S3. Logistic regression analysis of UHR for thrombi**

| **Variables** | **OR** | **95% CI** | ***P* value** |
| --- | --- | --- | --- |
| UA | 1.390 | 1.164-1.661 | <0.001 |
| HDL-C | 0.973 | 0.950-0.997 | 0.030 |
| LDL-C | 1.306 | 0.994-1.716 | 0.055 |
| UHR (continuous variable) | 1.087 | 1.039-1.136 | <0.001 |
| UHR (categorical variable) | 1.516 | 1.217-1.887 | <0.001 |
| **Model 1** | 1.360 | 1.070-1.729 | 0.012 |
| Q1 (4.77-10.25) | Reference |  |  |
| Q2 (10.26-13.64) | 2.061 | 1.006-4.222 | 0.048 |
| Q3 (13.65-17.39) | 3.183 | 1.562-6.486 | 0.001 |
| Q4 (17.40-38.83) | 3.625 | 1.763-7.454 | <0.001 |
| **Model 2** | 1.345 | 1.053-1.718 | 0.018 |
| Q1 (4.77-10.25) | Reference |  |  |
| Q2 (10.26-13.64) | 1.740 | 0.831-3.647 | 0.142 |
| Q3 (13.65-17.39) | 2.432 | 1.136-5.205 | 0.022 |
| Q4 (17.40-38.83) | 2.643 | 1.210-5.769 | 0.015 |
| **Model 3** | 1.348 | 1.053-1.727 | 0.018 |
| Q1 (4.77-10.25) | Reference |  |  |
| Q2 (10.26-13.64) | 2.851 | 1.183-6.875 | 0.020 |
| Q3 (13.65-17.39) | 2.916 | 1.187-7.164 | 0.021 |
| Q4 (17.40-38.83) | 3.072 | 1.211-7.795 | 0.018 |
| **Model 4** | 1.319 | 1.025-1.699 | 0.032 |
| Q1 (4.77-10.25) | Reference |  |  |
| Q2 (10.26-13.64) | 1.802 | 0.826-3.932 | 0.139 |
| Q3 (13.65-17.39) | 2.350 | 1.055-5.233 | 0.036 |
| Q4 (17.40-38.83) | 2.465 | 1.082-5.616 | 0.032 |

Model 1: UHR (categorical variable), age and gender.

Model 2: UHR (categorical variable), age, gender, atrial fibrillation, hypertension, diabetes mellitus and stroke.

Model 3: UHR (categorical variable), age, gender, atrial fibrillation, hypertension, diabetes mellitus, stroke, smoking and alcohol consumption.

Model 4: UHR (categorical variable), age, gender, atrial fibrillation, hypertension, diabetes mellitus, stroke, smoking, alcohol consumption, statins and UA-lowering drugs.

UA, uric acid; HDL-C, high-density lipoprotein cholesterol; LDL-C, low-density lipoprotein cholesterol; UHR, UA to HDL-C ratio; OR. odds ratio; CI, confidence interval.
